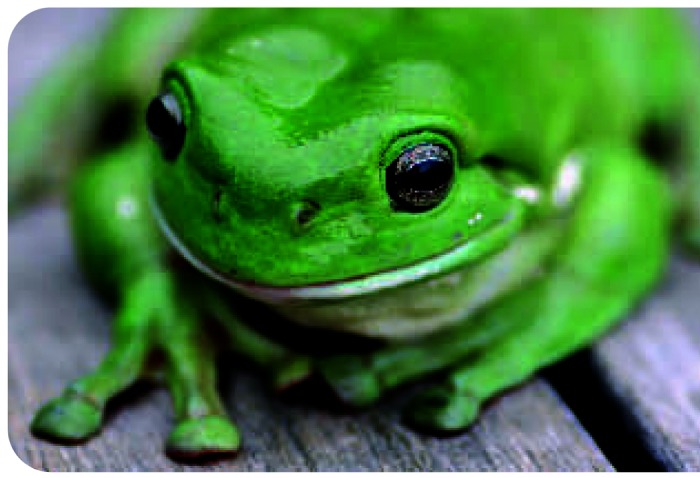# The Beat

**Published:** 2007-05

**Authors:** Erin E. Dooley

## Satellites for Health

A two-year National Research Council study released in January 2007 states that 17 new Earth-observing satellite missions need to be funded over the next decade if important data on climate change, hurricanes, and shifting drought and rainfall patterns are to be gathered. The landmark report sets forth the actions deemed most crucial by a 100-member panel of scientists for NASA and NOAA over the next 10 years. Panel cochair Richard Anthes said a $10 per capita investment in Earth-observing projects would pay for itself exponentially by improving weather forecasts, resource management, and hurricane preparedness. NASA’s budget for satellite measurements and analysis has been cut by more than 30% over the past five years.

**Figure:**
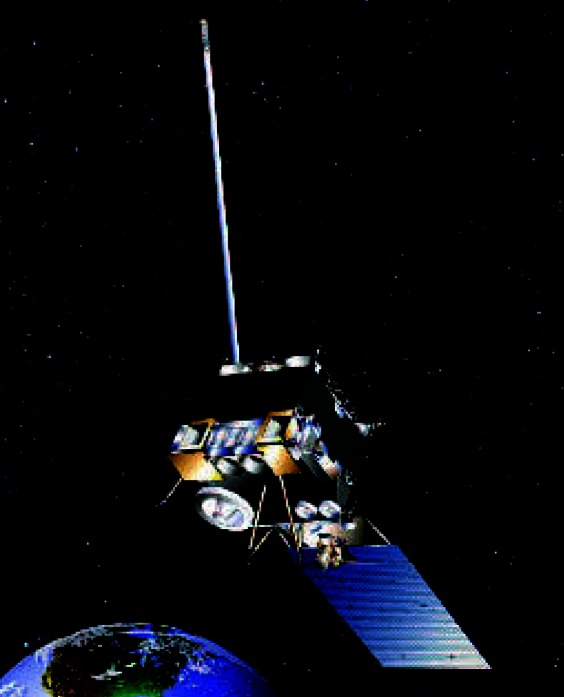


## Asia Buys In to Clean Energy

Two initiatives are under way to boost renewable energy sources in Asia. The first, in Nepal, calls for research into small-scale hydropower, biogas, and solar and wind power in an effort to reduce dependence on firewood, which negatively impacts indoor air quality, increases atmospheric pollution, and contributes to deforestation. In the second initiative, the leaders of 14 Asian countries, Australia, and New Zealand signed the Cebu Declaration on East Asian Energy Security in January 2007. The declaration calls for diversified energy sources, improved energy efficiency, and reduction of greenhouse gas emissions.

## Unsustainable Sustenance?

Low-birthweight babies are often fed a formula with extra nutrients and protein to help them catch up weight-wise and ensure they have adequate fat stores. A study in the 16 January 2007 issue of *Circulation* looked at newborns fed either fortified formula, regular formula, or breastmilk for the first nine months of their lives. At ages 6 to 8 years, the children fed enriched formula had significantly higher blood pressure than those in the other groups. The authors note that elevated blood pressure in childhood will probably carry over into adulthood and that lowering diastolic blood pressure at the population level by only 2 mm Hg could prevent nearly 100,00 heart attacks and strokes a year in the United States.

**Figure:**
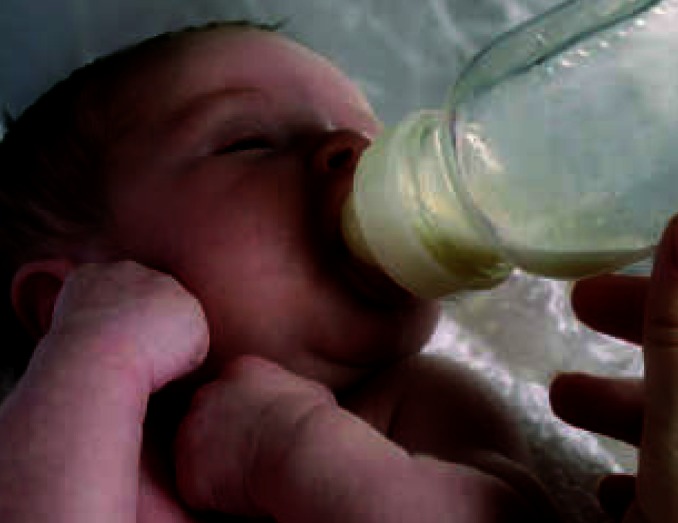


## Tortillas Take a Tumble in Mexico

Demand for the grain-based fuel ethanol is dramatically increasing international prices for corn, leading to a food crunch in Mexico, where tortilla prices have tripled or quadrupled since last summer. The 27 January 2007 *Washington Post* reports that lower-class Mexicans get more than 40% of their protein from tortillas, which also provide an important source of dietary fiber. The price increase threatens to result in a shift toward cheaper, less nutritious foods. In an effort to stem the price increases, Mexican president Felipe Calderón has instituted an agreement with business leaders to cap tortilla prices.

**Figure:**
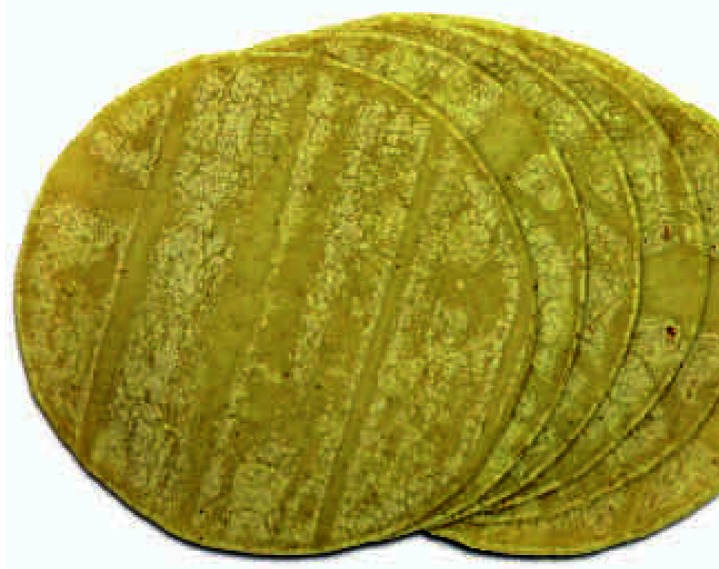


## Decline in Testosterone Levels

In a study notable for its large sample size and long duration, researchers from the New England Research Institutes report a substantial populationwide decline in testosterone levels over the past 20 years. The report on the Massachusetts Male Aging Study appears in the January 2007 issue of the *Journal of Clinical Endocrinology and Metabolism*. The decline appears unrelated to normal aging or to any health or environmental effects known to influence testosterone levels. Declines were measured at about 1.2% per year—about 17% overall between the years 1987 and 2004. Lower testosterone concentrations are linked with increases in age-related diseases, depression, and infertility.

## Amphibian Ark

Over the past decade, up to 170 species of frogs have become extinct due to the spread of *Chytrid* fungus, which affects amphibian respiratory and nervous systems. Close to 2,000 other species are threatened. In February 2007, the international Amphibian Ark project was launched in Atlanta, Georgia. The project is asking zoos, aquariums, and botanical gardens to house threatened frogs until the fungus can be controlled. Amphibians are a key component of ecosystems, eating insects that other animals do not. Amphibians also are medically important; various species produce compounds used in pharmaceuticals. Fundraising will commence next year to raise the estimated $400–500 million it will take to complete the project.

**Figure:**